# Clear aligner therapy: a scoping review of treatment prediction, clinical effectiveness, and limitations

**DOI:** 10.3389/fdmed.2026.1864529

**Published:** 2026-07-13

**Authors:** Ali Borzabadi-Farahani

**Affiliations:** Orthodontic Department, School of Dentistry, Cardiff University, Cardiff, United Kingdom

**Keywords:** anterior open bite, clear aligner, clear aligner therapy, extrusion, intrusion, overbite correction

## Abstract

**Background:**

Clear aligner therapy (CAT) has significant predictability limitations, particularly in complex malocclusions. This scoping review maps discrepancies between digitally predicted and clinically achieved tooth movements across a broad evidence base.

**Objectives:**

To map the available evidence on the accuracy of CAT tooth movements and identify gaps in the current literature to guide future research.

**Eligibility criteria:**

Studies evaluating the accuracy or predictability of CAT tooth movements using quantitative methods (intraoral scanning with 3D metrology, cone-beam computed tomography, or cephalometric analysis) were eligible. Eligible designs included retrospective and prospective cohort studies, randomised controlled trials, and systematic reviews, published from 2001 up to June 2026 with no language restriction. In-office aligner systems and non-proprietary aligner systems were excluded from this review.

**Sources of evidence:**

PubMed and Scopus were searched in April 2026 and updated in June 2026, supplemented by manual reference list searching.

**Charting methods:**

Data were extracted onto a pre-piloted standardised form. A descriptive narrative synthesis was performed, organised thematically by movement type.

**Results:**

Of 6,865 records retrieved after removal of duplicates, 764 were selected after screening, 356 underwent full-text review, and 119 studies met inclusion criteria, including 70 retrospective studies, 12 prospective studies, and 4 RCTs. Early-generation Invisalign studies report approximately 50% mean accuracy. Rotational shortfalls averaged approximately 5°; torque was under-expressed at 35%–58%; overbite reduction achieved 28%–55% accuracy; arch expansion (73%–88%) predominantly produced tipping rather than bodily movement. Anterior open bite correction reached 58%–90% accuracy, while incisor intrusion was limited to 33%–53%. Based on limited studies, refinements were required in over 50% of cases and 17.2% necessitated conversion to fixed appliances.

**Conclusions:**

Available evidence, predominantly retrospective Level III–IV studies, supports over-correction strategies of 50%–100% for intrusions, 5°–15° for rotations and torque, and 1–2 mm for arch expansions; however, these estimates derive from heterogeneous, Invisalign-focused research and should not be applied as universal benchmarks. CAT is most reliable for anterior open bite correction under 3 mm. Hybrid approaches and informed consent regarding potential fixed appliance conversion are advisable for complex malocclusions. Adequately powered RCTs are needed to validate current clinical protocols.

## 1 Introduction

Clear aligner therapy (CAT) represents a significant evolution in orthodontic treatment, providing patients with discreet, removable appliances for tooth alignment. The origins of CAT trace back to the 1940s, when Dr. Harold Kesling introduced the Tooth Positioner Appliance, a thermoformed retainer aimed at correcting minor incisor irregularities after removal of orthodontic appliances (1). By the late 1980s, the concept of sequential removable clear aligners emerged, culminating in the 1999 launch of Invisalign by Align Technology, which integrated computer-aided design and manufacturing (CAD/CAM) for precise planning and production.

Modern clear aligners are fabricated from transparent, biocompatible thermoplastic materials, typically polyethylene terephthalate glycol (PETG) or polyurethane-based laminates, offering aesthetic discretion, smooth surface texture that reduces soft tissue irritation, and ease of removal for oral hygiene maintenance. The material durability varies by system, with high-modulus laminates such as TC-85 and BENQ PET providing superior fatigue resistance over the wear cycle (113). These properties make CAT a preferable option for patients who prioritise aesthetics, have periodontal sensitivity, or require removability for contact sports or musical performance (2, 7).

**Figure 1:**
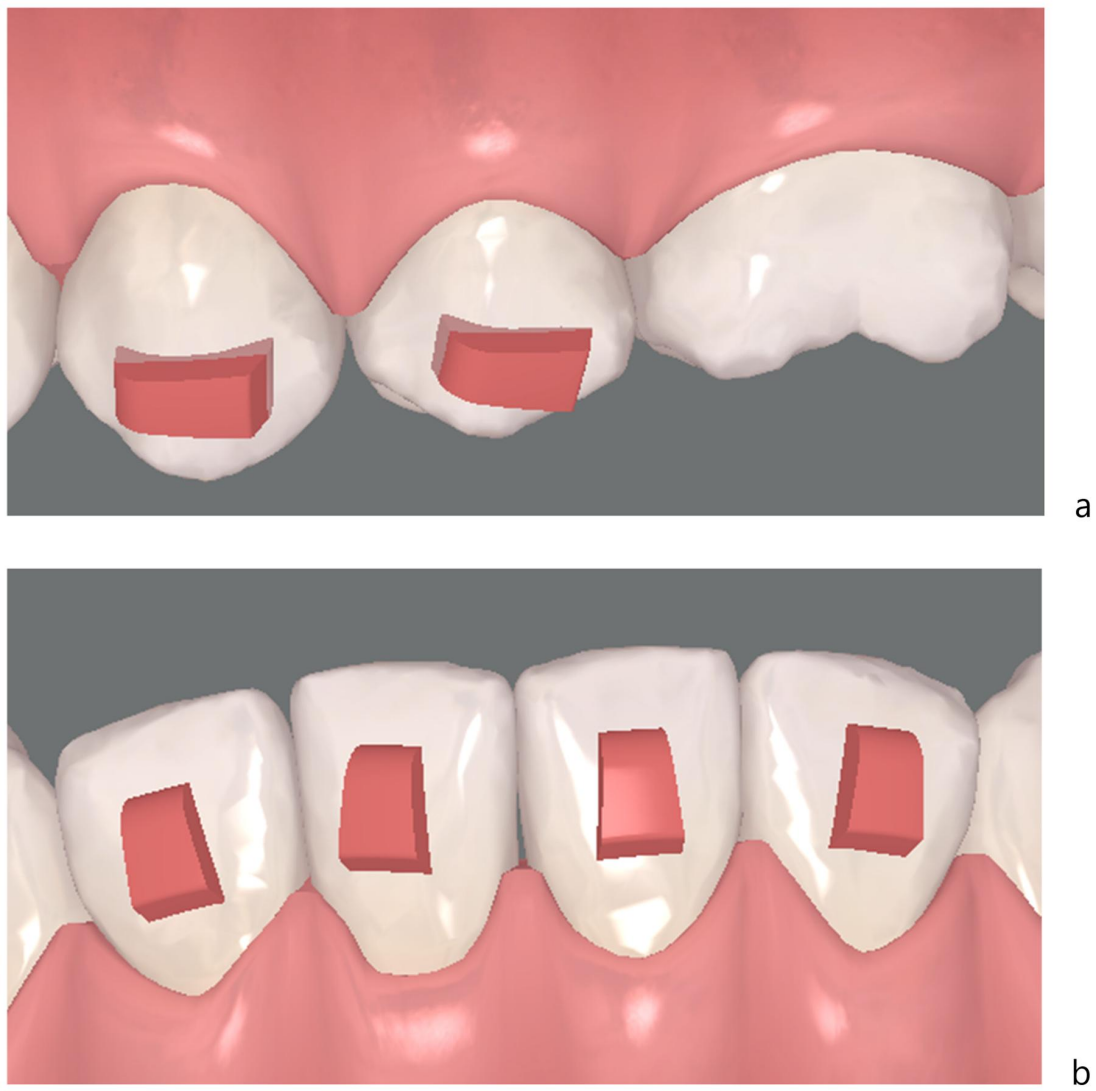
Illustration of two commonly used conventional attachments. **(a)** Rectangular horizontal attachment, designed for vertical movements, such as extrusion or open bite correction. **(b)** Rectangular vertical attachment, intended for horizontal movements, such as space closure.

Contrary to fixed orthodontic appliances, which primarily exert pulling forces on teeth, clear aligners apply pushing forces, allowing tooth movement until the tooth aligns with the aligner, with a standard maximum linear displacement of 0.25 mm per aligner in most systems (2). Technological advancements have significantly refined CAT, notably transitioning from traditional physical impressions to intraoral digital scans, which enhance both accuracy and efficiency. Attachments are pivotal in facilitating retention and enabling specific orthodontic movements. Conventional attachments, as illustrated in Figure 1, encompass ellipsoid designs for retention, rectangular horizontal attachments for vertical movements (e.g., open bite correction, premolar extrusion, and aligner retention on short clinical crowns), and rectangular vertical attachments for horizontal movements, such as distalization or space closure. Beveled attachments, configured in horizontal, vertical, or oblique (sash) orientations, are customized for specific movements, with gingivally beveled designs supporting extrusion, occlusally beveled designs facilitating intrusion, and sash configurations enabling simultaneous rotation and extrusion (3).

Optimized attachments, proprietary to Invisalign, encompass specialized variants designed for extrusion, root control, deep bite management, and multi-plane movements. For example, twin molar root control attachments generate force couples that facilitate bodily movement in extraction cases, thereby mitigating common side effects such as excessive molar tipping or incisor extrusion (2). Nevertheless, optimized attachments do not exhibit significant clinical advantages over conventional attachments (2, 3, 91, 142).

Treatment complexity substantially influences treatment planning, with patients frequently requiring nearly twice the number of aligners initially predicted (4). Extraction cases demand a greater number of aligners and more meticulous planning compared to non-extraction cases (5). Material properties, particularly the modulus of elasticity, significantly affect treatment outcomes: softer materials are suitable for simple alignments, whereas stiffer materials enable more complex movements, such as arch expansion, bite opening, and root movements (2). The aligner retention is primarily influenced by material properties such as modulus, elasticity, and fatigue resistance, rather than thickness alone, with high-modulus materials like TC-85 and BENQ PET offering the most consistent and effective performance (113).

**Figure 2:**
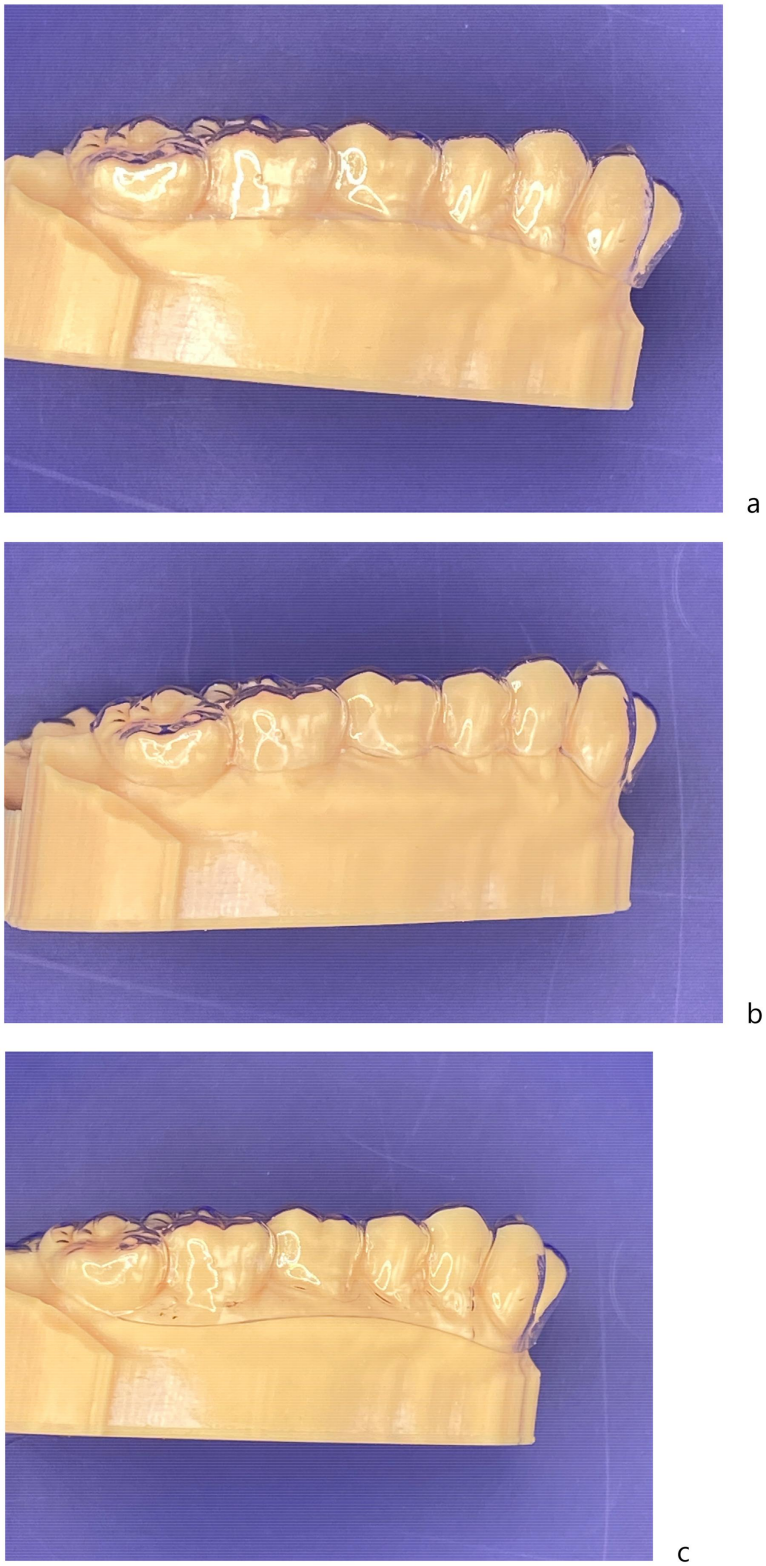
Different aligner trimlines; straight-cut at gingival level **(a)**, scalloped **(b)**, and straight-cut extended (2 mm) **(c)** It should be noted that this hierarchical classification of trimline designs is derived from a single laboratory-based study (6) and has not yet been validated in prospective clinical trials. Clinicians should apply this guidance cautiously pending further *in-vivo* evidence.

Aligner trimlines, whether straight or scalloped, profoundly influence force delivery, with straight-cut extended designs offering superior retention compared to scalloped designs (6) (Figure 2). A laboratory-based study by Elshazly et al. (6) proposes the following hierarchical order of efficacy for aligner trimline designs (Table 1);

**Table 1:** Hierarchical order of efficacy for different aligner trimlines according to the lab study by Elshazly et al. (6).

Rank	Trimline design	Key characteristics
1	Straight-cut extended (2 mm)	No attachments required for retention
2	Straight-cut at gingival level	–
3	Scalloped	–

Altered aligner geometries, such as anterior bite ramps and power ridges incorporated into aligners, are designed to enhance torque expression (e.g., lingual or palatal root movement in incisors) and facilitate bite opening (e.g., overbite correction). However, evidence supporting their efficacy in achieving overbite reduction or effective torque delivery remains limited (2).

Adjunctive fixed appliances are frequently integrated into the treatment of complex cases, with approximately 50% of orthodontists in Canada reporting their use for such scenarios (7). This scoping review examines the difference between virtual treatment plans (software simulations or predicted amount) and achieved or actual clinical outcomes in CAT, highlighting key achievements, limitations, and strategies for optimization. Given the heterogeneity of study designs, aligner systems, and outcome measures in this field, a scoping review methodology was selected to comprehensively map the available evidence rather than synthesise a single focused clinical question. This approach allows for a broad mapping of movement accuracy, overcorrection strategies, and current clinical practices across heterogeneous evidence.

## 2 Methodology

**Figure 3:**
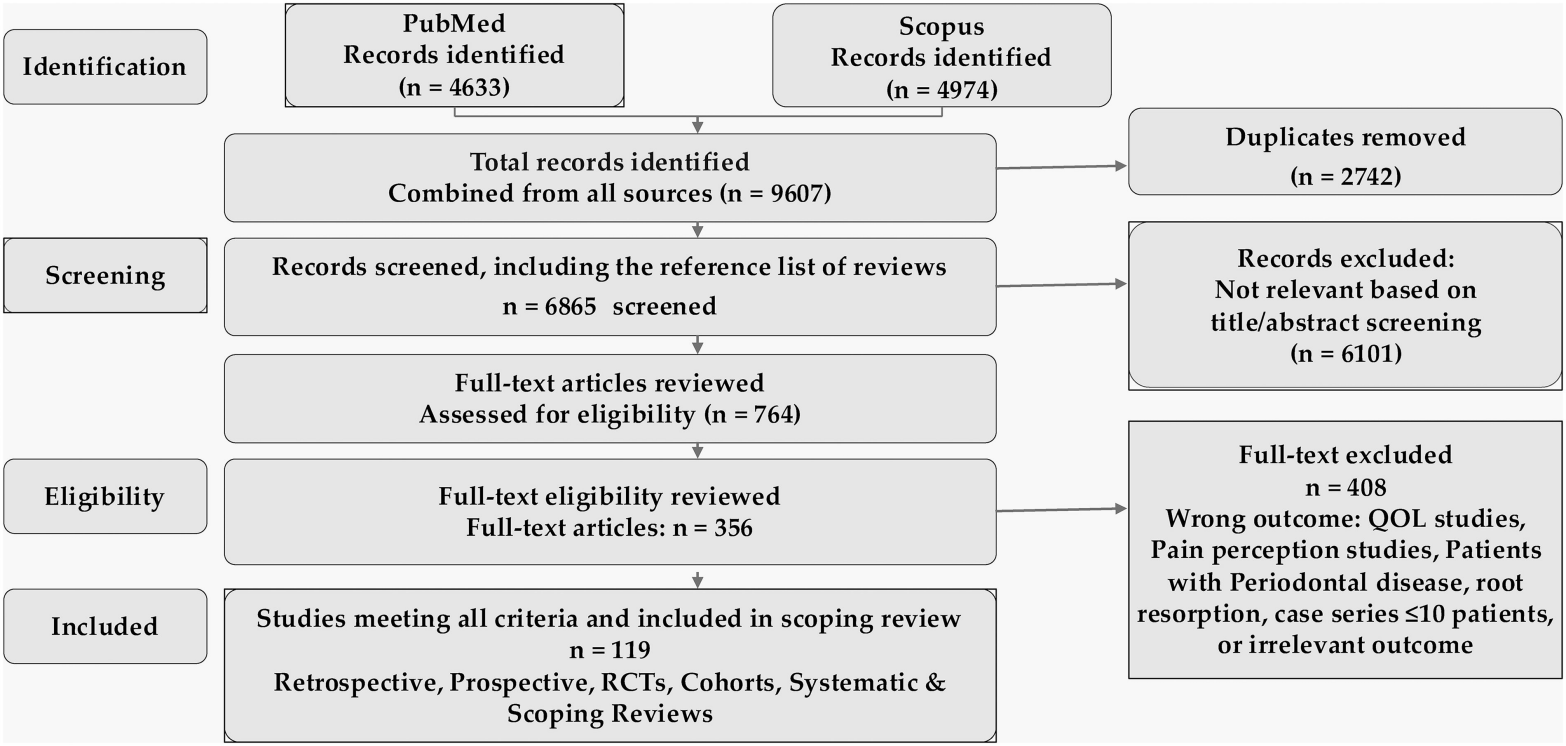
The PRISMA flowchart for the present scoping review.

This scoping review was conducted in accordance with the framework described by Levac et al. (115) and is reported following the PRISMA-ScR checklist (116). A structured literature search was conducted in PubMed and Scopus, due to their strong coverage of dental/orthodontic literature, in April 2026 and updated in June 2026, with no language restrictions. Manual searching of reference lists and grey literature sources was also performed. The search covered publications from 2001 upto June 2026 (Figure 3).

### 2.1 Population-concept- context framework, databases and search strategy

The following Population, Concept, and Context framework was applied:

**Population:** Adults and adolescents undergoing orthodontic treatment
**Concept:** Predictability and accuracy of clear aligner therapy tooth movements
**Context:** Clinical orthodontic setting

The wide PubMed search string was: [(MeSH Terms and free-text keywords as listed below)]. Duplicate records were identified and removed. One reviewer (ABF) independently screened titles and abstracts, followed by full-text review.

Key databases were implicitly searched, with an emphasis on Invisalign due to its prominence in the literature, alongside other aligner systems where relevant. For instance, the PubMed searches used following keywords and MeSH Terms:

#### 2.1.1 Population/appliance type

**MeSH:** “Malocclusion, Angle Class I”[MeSH], “Malocclusion, Angle Class II”[MeSH], “Malocclusion, Angle Class III”[MeSH], “Orthodontic Appliances, Removable”[MeSH], “Orthodontic Appliances, Fixed”[MeSH], “Orthodontics, Corrective”[MeSH].

**Free-text:** “clear aligner”, “clear aligner therapy”, “Invisalign”, “thermoplastic aligner”, “orthodontic aligner”, “transparent aligner”, “removable appliance”, “fixed appliance”, “braces”.

#### 2.1.2 Concept (tooth movement/accuracy)

**MeSH:** “Tooth Movement Techniques”[MeSH], “Treatment Outcome”[MeSH].

**Free-text:** “predictability”, “prediction accuracy”, “predictive error”, “simulation accuracy”, “movement accuracy”, “tooth movement”, “torque”, “extrusion”, “intrusion”, “expansion”, “overbite”, “occlusal contact”, “curve of Spee”, “achievement”.

#### 2.1.3 Context (clinical setting/methods)

**MeSH:** “Cone-Beam Computed Tomography”[MeSH], “Cephalometry”[MeSH], “Imaging, Three-Dimensional”[MeSH].

**Free-text:** “digital treatment planning”, “computer simulation”, “software simulation”, “intraoral scan”, “3D superimposition”, “CBCT”, “cephalometric analysis”, “3D metrology”, “clinical outcome”, “final occlusion”.

Subsequently, using a combination of controlled vocabulary, synonyms, text words/search terms, Boolean operators, and different translations, as the following wide search string was used for Pubmed:

(“malocclusion, angle class i”[MeSH Terms] OR “malocclusion, angle class ii”[MeSH Terms] OR “malocclusion, angle class iii”[MeSH Terms] OR “orthodontic appliances, removable”[MeSH Terms] OR “orthodontic appliances, fixed”[MeSH Terms] OR “orthodontics, corrective”[MeSH Terms] OR “clear aligner”[Title/Abstract] OR “clear aligner therapy”[Title/Abstract] OR “Invisalign”[Title/Abstract] OR “thermoplastic aligner”[Title/Abstract] OR “orthodontic aligner”[Title/Abstract] OR “transparent aligner”[Title/Abstract] OR “removable appliance”[Title/Abstract] OR “fixed appliance”[Title/Abstract] OR “braces”[Title/Abstract]) AND (“Tooth Movement Techniques”[MeSH Terms] OR “Treatment Outcome”[MeSH Terms] OR “predictability”[Title/Abstract] OR “prediction accuracy”[Title/Abstract] OR “predictive error”[Title/Abstract] OR “simulation accuracy”[Title/Abstract] OR “movement accuracy”[Title/Abstract] OR “tooth movement”[Title/Abstract] OR “torque”[Title/Abstract] OR “extrusion”[Title/Abstract] OR “intrusion”[Title/Abstract] OR “expansion”[Title/Abstract] OR “overbite”[Title/Abstract] OR “occlusal contact”[Title/Abstract] OR “curve of Spee”[Title/Abstract] OR “achievement”[Title/Abstract]) AND (“Cone-Beam Computed Tomography”[MeSH Terms] OR “Cephalometry”[MeSH Terms] OR “imaging, three dimensional”[MeSH Terms] OR “digital treatment planning”[Title/Abstract] OR “computer simulation”[Title/Abstract] OR “software simulation”[Title/Abstract] OR “intraoral scan”[Title/Abstract] OR “3D superimposition”[Title/Abstract] OR “CBCT”[Title/Abstract] OR “cephalometric analysis”[Title/Abstract] OR “3D metrology”[Title/Abstract] OR “clinical outcome”[Title/Abstract] OR “final occlusion”[Title/Abstract]) AND (“2001/01/01”[Date—Publication]: “3000”[Date—Publication]).

The following wide search string was used for Scopus:

[TITLE-ABS-KEY (“malocclusion” OR “angle class i” OR “angle class ii” OR “angle class iii” OR “clear aligner” OR “clear aligner therapy” OR “invisalign” OR “thermoplastic aligner” OR “orthodontic aligner” OR “transparent aligner” OR “removable appliance” OR “fixed appliance” OR “braces”)] AND [TITLE-ABS-KEY (“tooth movement” OR “treatment outcome” OR “predictability” OR “prediction accuracy” OR “predictive error” OR “simulation accuracy” OR “movement accuracy” OR “torque” OR “extrusion” OR “intrusion” OR “expansion” OR “overbite” OR “occlusal contact” OR “curve of spee” OR “achievement”)] AND [TITLE-ABS-KEY (“cone beam computed tomography” OR “cephalometry” OR “digital treatment planning” OR “computer simulation” OR “software simulation” OR “intraoral scan” OR “3d superimposition” OR “cbct” OR “cephalometric analysis” OR “3d metrology” OR “clinical outcome” OR “final occlusion”)] AND PUBYEAR > 2,000.

### 2.2 Eligibility criteria and data extraction

Inclusion criteria included studies (cohort, clinical trial, RCT, scoping or systematic reviews as well as narrative reviews with structured search strategy) evaluating movement accuracy through tools such as intraoral scans and relevant (3D) metrology software, cone-beam computed tomography (CBCT), or cephalometric analysis. The accuracy percentage can be calculated as (actual amount/predicted amount) × 100%. The findings of relevant systematic reviews have been included. The exclusion criteria were all studies that didn't investigate the clear aligner therapy, case reports, case series with ≤10 patients, opinion pieces, editorials, lab studies without clinical relevance. In-office aligner systems and non-proprietary aligner systems were excluded from this review. This scoping review employs a qualitative synthesis, prioritizing clinical relevance. A descriptive/narrative synthesis was performed. No meta-analysis was conducted due to the heterogeneity of study designs, outcome measures, and aligner systems. Findings are presented thematically by movement type. This scoping review was not prospectively registered in PROSPERO.

To enhance transparency, the complete study selection process is depicted in the PRISMA-ScR flowchart (Figure 3). Eligibility assessment was performed by a single reviewer (ABF) across two independent screening rounds separated by a two-week interval, using a standardised eligibility form. Data were extracted onto a pre-piloted data extraction table, capturing study design, aligner system, sample characteristics, movement types assessed, and accuracy metrics. In keeping with scoping review methodology, no formal risk-of-bias tool was applied; methodological limitations of included studies are discussed narratively in the Evidence Gaps section.

The following variables were extracted for each study: (1) study design; (2) aligner system; (3) sample size and age group; (4) extraction vs. non-extraction protocol; (5) movement type(s) assessed; (6) study objectives and accuracy measurement method; (7) accuracy (%); (8) control or comparison group.

## 3 Results

A total of 9,607 records were identified through database searching, including 4,633 records from PubMed and 4,974 records from Scopus. After removal of 2,742 duplicate records, 6,865 unique records remained for title and abstract screening. Records underwent title and abstract screening and relevant records identified (*n* = 764). Of these, 356 were retrieved for full-text review, and finally 119 studies met the inclusion criteria and were included in the evidence synthesis (1–5, 8, 10, 13–16, 19–68, 71, 76–78, 80–83, 85–99, 101–105, 107–114, 119, 120, 127–140, 142–145, 147, 148).

### 3.1 Characteristics of included studies

The reviewed literature revealed several methodological challenges, including predominantly retrospective observational designs, small sample sizes, lack of sample size estimation (8), absence of randomization, single-clinician bias, and assumptions regarding centres of resistance due to limited root length information. Sub-group analyses are very scarce examining the roles of auxiliaries, over-corrections, patient compliance, and non-Invisalign systems. There is apparent shortage of randomized clinical trials in the CAT field (8).

The 119 included studies were categorised into three broad groups:

1. Primary studies directly evaluating CAT accuracy and outcomes (*n* = 72, Table 2) (3–5, 13–15, 19, 21–34, 36, 38–53, 55, 76, 77, 83, 87, 89, 92, 95–97, 101, 102, 104, 107, 109, 110, 119, 120, 128, 130, 132–140, 142–145, 147).
2. Comparative studies evaluating CAT vs. fixed appliance therapy (*n* = 14, Table 3) (20, 54, 56, 58, 59, 67, 68, 94, 103, 105, 112, 129, 131, 148).
  These two categories form 70 retrospective studies, 12 prospective studies, and 4 RCTs.
3. The secondary evidence including systematic reviews, meta-analyses, narrative reviews, and consensus documents (*n* = 33) (1, 2, 8, 10, 16, 35, 37, 57, 60–66, 71, 78, 80–82, 85, 86, 88, 90, 91, 93, 98, 99, 108, 111, 113, 114, 127).

**Table 2:** CAT primary clinical studies and summary of study characteristics. Sample sizes accompanied by key inclusion criteria were reported. Relevant criteria include age group (adult/adolescent), dentition stage, extraction versus non-extraction protocol, and primary malocclusion type.

Author (Year)	Study type	Aligner type	Sample size	Control/comparison group
			Inclusion criteria	
			Assessments	
1-Clements et al. (2003) (128)	Prospective study	Invisalign	51 adults Different malocclusions, extraction and non-extraction treatment 1-week activation with soft material *Assessed the effect of wearing protocol (1 week or 2 weeks) and using hard or soft aligners using little's incisor irregularity index and PAR*.	1-week activation with hard material, 2-week activation with soft material, and 2-week activation with hard material
2-Baldwin et al. (2008) (130)	Prospective study	Invisalign	24 adults Different malocclusions, all had at least 1 premolar extracted *Assessed the movement of teeth adjacent to premolar extraction spaces during space closure.*	–
3-Kravitz et al. (2008) (40)	Prospective study	Anterior Invisalign	31 adults 2–3 weeks wear protocol *Assessed the influence of attachments and interproximal reduction on canine rotation.*	–
4-Kravitz et al. (2009) (13)	Prospective study	Anterior Invisalign	37 adults Anterior crowding or spacing (<5 mm) and adequate buccal interdigitation *Assessed the efficacy of tooth movement.*	–
5-Krieger et al. (2012) (132)	Retrospective study	Invisalign	50 patients Aged 15–63 years Anterior crowding in the maxilla and/or mandible *Assessed the predicted movement in the anterior region.*	–
6-Kassas et al. (2013) (133)	Retrospective study	Invisalign	31 patients Aged 35.2 ± 13.2 years Non-extraction treatment *Assessed the changes in model grading system score of the ABO (similar to ABO Objective Grading System Score without root angulation assessment).*	–
7-Chisari et al. (2014) (143)	Prospective CBCT clinical trial	Invisalign	30 adults Minor incisor malalignments Group I: 15 patients, 18–35 years *Assessed the OTM on CBCT for the right or left maxillary central incisor.*	Group II:15 patients ≥50 years
8-Simon et al. (2014) (134)	Retrospective study	Invisalign	26 patients Aged 13–72 years *Assessed the influence of auxiliaries (Attachment/Power Ridge) and the staging (movement per aligner) on incisor torque, premolar derotation and molar distalization.*	–
9-Houle et al. (2017) (50)	Retrospective study	Invisalign	64 adults Mean age = 31.2 years (18 to 61 years) 20 had at least one crossbite *Assessed maxillary arch changes.*	–
10-Khosravi et al. (2017) (28)	Retrospective cephalometric study	Invisalign	120 patients Median age = 33 years 68 patients with normal overbites, 40 with deepbites, and 12 with open bites *Assessed the OB correction mechanism.*	–
11-Moshiri et al. (2017) (22)	Retrospective cephalometric study	Invisalign	30 adults Had AOB *Assessed the vertical effects of non-extraction treatment of AOB.*	–
12-Lombardo et al. (2017) (144)	Retrospective study	F22 aligners	16 adults 2-week wear protocol *Assessed the predictability of CAT in guiding teeth into the planned positions.*	–
13-Tepedino et al. (2018) (145)	Retrospective study	Nuvola® aligners	39 adults Mean age = 30.7 years Non-extraction treatment anterior crowding (≤6 mm) 2-week wear protocol *Assessed the predictability of CAT in predicted tooth movement.*	–
14-Charalampakis et al. (2018) (76)	Retrospective study	Invisalign	20 adults Class I *Assessed the accuracy of specific tooth movements.*	–
15-Dai et al. (2019) (15)	Retrospective study	Invisalign	30 patients Aged 19.4 ± 6.3 years 15 patients had Class I, 11 Class II, and 4 had Class III molar occlusions Had maxillary first premolar extraction *Assessed tooth movements of maxillary first molars and central incisors in first premolar extraction cases.*	–
16-Haouili et al. (2020) (14)	Prospective study	Invisalign Full or Invisalign Teen	38 adults 22 Class I, 13 Class II, and 3 Class III malocclusion patients *Assessed the accuracy of tooth movement with Invisalign.*	–
17-Harris et al. (2020) (23)	Retrospective study	Invisalign	45 adults *Assessed the efficacy of AOB correction.*	–
18-Morales-Burruezo et al. (2020) (51)	Retrospective study	Invisalign (SmartTrack)	114 adults *Assessed the efficacy of arch expansion.*	–
19-Zhou & Guo (2020) (137)	Retrospective CBCT study	Invisalign	20 adults *Assessed the efficiency of bodily expansion in upper arch expansion*	–
20-Al-Nadawi et al. (2021) (26)	Retrospective study	Invisalign (SmartTrack)	80 adults 7-day changes (*n* = 27) *Assessed the effect of wear protocol.*	10-day changes (*n* = 27) 14-day changes (*n* = 26)
21-Karras et al. (2021) (3)	Retrospective cohort study	Invisalign	100 patients aged 11–63 years Majority were Class I malocclusions *Assessed the effect of optimized and conventional attachments on rotational and extrusive tooth movements.*	–
22-Patterson et al. (2021) (27)	Retrospective study	Invisalign	80 adults Class I (*n* = 40), Class II (*n* = 40) *Assessed the efficacy of Class II malocclusion correction.*	–
23-Blundell et al. (2021) (32)	Retrospective study	Invisalign (SmartTrack)	42 adults Deep bite, non-extraction treatment *Assessed the accuracy of deep overbite correction.*	–
24-Jiang et al. (2021) (43)	Retrospective CBCT study	Clear aligners (not specified)	69 adults *Assessed the efficacy of different types of incisor movements (e.g., pure tipping and torque).*	–
25-Gaddam et al. (2021) (44)	Retrospective study	Invisalign	40 adults Simple Class I malocclusion comprising spacing (<4 mm) to crowding (<6 mm) Non-extraction treatment, Two-week wear protocol *Assessed the accuracy of torque expression.*	–
26-Dai et al. (2021) (139)	Retrospective study	Invisalign	17 adults 12 had Class I occlusion, 4 with cusp-to-cusp Class II occlusions, and 1 with a cusp-to-cusp Class III molar occlusion *Assessed the achieved and predicted crown movements of first molars, canines, and central incisors after 4 first premolar extraction treatment with CAT.*	–
27-Graf et al. (2021) (147)	Retrospective study	Invisalign	33 adults 7 Class I, 24 Class II and 2 Class III patients *Assessed the treatment effectiveness of CAT and the stability of these effects after a short-term retention period using the PAR Index.*	–
28-Blundell et al. (2022) (33)	Retrospective study	Invisalign (SmartTrack vs EX30)	68 adults Non-extraction treatment 2-weekly wear protocol Newer SmartTrack material and precision bite ramps (*n* = 39) *Assessed the effect of using bite ramps on deep overbite correction.*	EX30 material with no bite ramps (*n* = 29)
29-Maree et al. (2022) (41)	Retrospective study	Invisalign	30 adults *Assessed the efficacy of rotation and uprighting tooth movements of bilateral winged maxillary central incisors.*	–
30-Stephens et al. (2022) (42)	Retrospective cohort study	Invisalign (SmartTrack)	75 Adults Optimized rotation attachments, 1-week protocol (*n* = 25) *Assessed the effect of optimized rotation attachments.*	Optimized rotation attachments, 2-week wear protocol (*n* = 25) Conventional nonbeveled rectangular attachments, 2-week wear protocol (*n* = 25)
31-Talens-Cogollos et al. (2022) (19)	Retrospective cephalometric study	Invisalign	58 adults Skeletal Class I *Assessed the unplanned molar intrusion.*	–
32-Suh et al. (2022) (24)	Retrospective cephalometric study	Invisalign	69 adults AOB patients, non-extraction treatment Angle's Class I, II, and III groups Skeletal AOB (*n* = 50) Dental AOB (*n* = 19) *Assessed the effective ness of AOB correction.*	–
33-Goh et al. (2022) (38)	Retrospective study	Invisalign	42 adults (majority) Class I or II Angle malocclusions *Assessed the mandibular CoS levelling*	–
34-Smith et al. (2022) (47)	Retrospective study	Invisalign	42 adults Class I occlusion with minimal anterior–posterior movement planned; mild crowding, non-extraction treatment *Assessed the lower incisor tip and root with vertical attachment.*	–
35-Fiori et al. (2022) (135)	Retrospective study	Invisalign	40 adults Severe crowding in both arches Non-extraction treatment *Assessed the predictability of crowding resolution and the efficacy of transversal arch expansion, arch length, and IPR to gain space.*	–
36-Taffarel et al. (2022) (136)	Retrospective study	Invisalign	32 adults Class II malocclusion *Assessed whether the CAT of Class II malocclusion with sequential distalization of posterior teeth would meet the ABO Model Grading System.*	–
37-D'Antò et al. (2022) (138)	Prospective study	Invisalign	17 adults Mean age = 28.3 years Non-extraction treatment Crowding and spacing <7 mm per arch Absence of tooth shape anomalies, supernumerary teeth, and tooth rotation more than 35° *Assessed the virtually planned and the achieved tooth movement (torque, tip and rotation).*	–
38-Feng et al. (2022) (140)	Retrospective CBCT study	Invisalign	21 adults Mean age = 29.5 ± 5.2 years All had first premolar extraction and masse retraction of the maxillary anterior teeth *Assessed the designed and achieved mesiodistal angulation of maxillary canines and posterior teeth for first premolar extraction with CAT and identify the main influencing factors for preventing tooth tipping toward the extraction space.*	–
39-Meade et al. (2023) (4)	Retrospective cohort	Invisalign	324 adults Records were from 11 orthodontist Non-extraction treatment *Assessed the total number of initial digital treatment plan and refinements.*	–
40-Bowman et al. (2023) (31)	Retrospective study	Invisalign	33 adults Mild-to-moderate Class I malocclusions *Assessed occlusal contacts, overbite, transverse expansion, and the buccolingual inclination of the teeth.*	–
41-Bowman et al. (2023) (55)	Retrospective study	Invisalign	33 adults Mild-to-moderate Class I malocclusions (spacing <4 mm or crowding of <6 mm) *Assessed the occlusal contact outcomes.*	–
42-Blundell et al. (2023) (25)	Retrospective study	Invisalign	76 adults Non-extraction treatment Open bite of one or both maxillary central incisors *Assessed the correction of AOB.*	–
43-Lim et al. (2023) (39)	Retrospective study	Invisalign	53 adults Non-extraction treatment Class I or II malocclusions *Assessed the leveling the maxillary* CoS	–
44-Kravitz et al. (2023) (83)	Retrospective study	Invisalign	500 patients Mean age = 33.6 years 75% Class I, 20% Class II, 5% Class II *Assessed the number of refinements and percentage of patients switched from Invisalign to braces to finish treatment*	–
45-Suh et al. (2023) (77)	Retrospective study	Invisalign	52 adults AOB patients *Assessed the stability of AOB treatment.*	–
46-Galluccio et al. (2023) (52)	Retrospective study	Invisalign	28 patients mean age = 17 ± 3.2 yrs non-extraction treatment *Assessed maxillary arch transverse expansion.*	–
47-Allahham et al. (2023) (87)	Retrospective CBCT study	Invisalign	29 adults Skeletal Class I or mild Class II relationship Mild-to-moderate pretreatment crowding Non-extraction *Assessed alveolar bone dehiscences and fenestrations post treatment.*	–
48-Shahabuddin et al., (2023) (119)	Retrospective study	Invisalign	24 adults 17 Angle Class I malocclusion, 7 Class II at least on 1 side ≥4 mm or ≥50% pre-treatment overbite *Assessed the efficacy of deepbite correction.*	–
49-Castroflorio et al. (2023) (142)	Prospective study	Invisalign	79 adults Mean age = 30.8 years Class I or mild Class II malocclusion Mild-to-moderate crowding or spacing *Assessed which are the less predictable OTM with CAT and if the presence and shape of attachment (no attachment, conventional or optimized attachments) or wear protocol (7 or 14 days) influence the predictability of OTM.*	–
50-Meade & Weir (2024) (5)	Retrospective study	Invisalign	355 adults 71.5% non-extraction, 28.5% had extractions 33% had OJ and OB > 4 mm *Assessed the efficacy of overjet and overbite correction.*	–
51-Blundell et al. (2024) (29)	Retrospective study	Invisalign	102 adolescents Aged 10–17 years Class I molar relationship or within 2 mm of a Class I molar relationship *Assessed the efficacy of deep overbite correction.*	–
52-Kravitz et al. (2024) (30)	Prospective study	Invisalign Teen or Invisalign Full	58 patients Adolescents (*n* = 29) *Assessed the efficacy of deep overbite correction.*	Adults (*n* = 29)
53-Rajan et al. (2024) (45)	Retrospective study	Invisalign	63 adults Non-extraction 1 week wear protocol (*n* = 49) *Assessed the lingual root torque of the maxillary central incisors.*	2 weeks wear protocol (*n* = 14)
54-Thilagalavanian et al. (2024) (48)	Retrospective study	Invisalign	54 adults Had U4 or U5 extracted *Assessed the root angulation in canine, premolar, and first molar teeth adjacent to first and second premolar extraction sites in the maxilla.*	–
55-Loberto et al. (2024) (53)	Retrospective study	Invisalign	36 patients aged 8.3 ± 1.5 years Posterior transverse discrepancy between maxillary and mandibular arches up to 6 mm, with or without cross-bite *Assessed the maxillary and mandibular expansion in mixed dentition.*	–
56-Basudan et al. (2025) (109)	Retrospective CBCT study	Invisalign	30 adults Non-extraction cases 10 days wear protocol *Assessed maxillary transverse and anteroposterior movements.*	–
57-Fialho et al. (2025) (104)	Randomised Clinical Trial	Invisalign	50 adults Class I malocclusions with mild crowding 12 h wear-time protocol (*n* = 25) *Assessed the effect of wear protocol on treatment outcomes.*	22 h wear protocol (*n* = 25)
58-Wu et al. (2025) (110)	Retrospective CBCT study	Clear aligners (not specified)	38 patients Mean age = 9.2 years Deep overbite patients Divided into 3 groups of 16 at different mixed dentition Demirjian stages (F, G, H) *Assessed the effect of different mixed dentition Demirjian stages on deepbite correction.*	Control group (*n* = 46) without any orthodontic treatment
59-Chan et al. (2025) (21)	Retrospective cohort	Invisalign	83 adults Had extraction of only one lower incisor *Assessed the achieved tooth movements (OJ, OB, labiolingual inclination) and the prevalence of open gingival embrasures.*	–
60-Han et al. (2025) (36)	Retrospective study + CBCT sub-sample	Invisalign	33 adult females CBCT sub-sample (*n* = 9) Skeletal class I Deep overbite (overbite ≥4 mm or ≥40%) *Assessed the accuracy of incisor intrusion in* *non-extraction deep overbite cases.*	–
61-Jin et al. (2025) (34)	Retrospective study	Invisalign	24 patients Mostly adults with deepbite (≥ 50%) Non-extraction treatment *Assessed the clinical outcomes of anterior teeth intrusion over-correction with the reverse CoS on aligner.*	–
62-Rajan et al. (2025) (46)	Retrospective cohort	Invisalign	35 adults Non-extraction treatment *Assessed the achieved mandibular central incisor lingual root torque.*	–
63-Thilagalavanian et al. (2025) (49)	Retrospective study	Invisalign	31 adults Had extraction of L4 or L5 *Assessed planned root angulation of molars, premolars, and canines adjacent to mandibular premolar extraction sites.*	–
64-Tang et al. (2025) (92)	Retrospective study	Invisalign	60 adults Non-extraction of mandibular teeth or extraction of bilateral mandibular 1st premolars Extraction group (*n* = 30) *Assessed leveling of CoS in cases with extractions.*	Non-extraction group (*n* = 30)
65-Burns et al. (2025) (89)	Retrospective study	Invisalign	48 Children (*n* = 30), Adults (*n* = 18) Skeletal Class I or mild to moderate Class II/III malocclusions, mild to moderate dental crowding (<5 mm), and deep OB *Assessed mandibular incisor intrusion.*	–
66-Hatami et al. (2026) (107)	Retrospective study	Invisalign (SmartTrack)	51 adults Class II subdivision malocclusion *Assessed the outcome of unilateral Class II molar correction.*	–
67-Cremonini et al. (2026) (102)	Retrospective cohort study	F22 aligners	40 adults Class I dental malocclusion with minimal crowding (≤ 3 mm) Hybrid (*n* = 20), aligners and segmental lingual appliance *Assessed managing severe rotation of maxillary and mandibular canines and premolars using hybrid approach (a segmental lingual appliance).*	Non-hybrid (*n* = 20) aligner and rotation attachments
68-Meade et al. (2026) (97)	Retrospective study	Invisalign	199 Adolescents Class II molar relationships Non-extraction *Assessed the efficacy of Class II corrections with CAT + elastics.*	–
69-Meade et al. (2026) (101)	Retrospective analysis	Invisalign Lite	122 adults *Assessed the planned changes in OJ and OB.*	–
70-Xiang et al. (2026) (95)	Retrospective CBCT study	Angel aligner	20 adults Non-extractions Class I or mild II Angle malocclusion with OB ≥ 4 mm *Assessed the efficacy of mandibular anterior intrusion.*	–
71- Kou et al. (2026) (120)	Retrospective analysis	Smartee® system	36 adults Absence of crowding in the anterior teeth with deep overbite BitePlane group (*n* = 15) *Assessed the effect of bite planes incorporated into upper aligners on anterior intrusion and posterior displacement during deep overbite correction.*	Control group (*n* = 21)
72-Issa et al. (2026) (96)	Retrospective study	Clear aligners (not specified)	29 adults IOTN score of 2 or 3 Single attachment group (*n* = 14) *Assessed the effectiveness of single vs. double attachments for correction of premolar rotation.*	Two attachments group (*n* = 15)

**Table 3:** Compar*ative CAT and FAT clinical studies and* summary of study characteristics. Sample sizes accompanied by key inclusion criteria were reported. Relevant criteria include age group (adult/adolescent), dentition stage, extraction versus non-extraction protocol, and primary malocclusion type.

Author (Year)	Study type	Aligner type	Sample size	Control/comparison group
			Inclusion criteria	
			Assessments	
1-Djeu et al. (2005) (54)	Retrospective cohort study	Invisalign	96 adults Non-extraction CAT (*n* = 48) *Assessed the changes in ABO Objective Grading System Score (alignment, marginal ridges, buccolingual inclination, occlusal contacts, occlusal relations, overjet, interproximal contacts, and root angulation).*	FAT (*n* = 48), treated with Tip-Edge system
2-Kuncio et al. (2007) (129)	Retrospective study	Invisalign	22 adults Non-extraction CAT (*n* = 11) *Assessed Objective Grading System Score at the end of treatment and post retention.*	FAT (*n* = 11), treated with Tip-Edge system
3-Pavoni et al. (2011) (131)	Prospective study	Invisalign	40 adults Class I malocclusion with mild crowding CAT (*n* = 20) Mean age of 18 years 4 months *Assessed the changes in the transverse dimension and the perimeter of the maxillary arch.*	FAT (*n* = 20), treated with self-ligating TIME 3 Mean age of 15 years 6 months
4-Garnett et al. (2019) (59)	Retrospective study	Invisalign	53 adults Hyperdivergent AOB patients (mandibular plane angles of ≥38°) CAT (*n* = 36) *Assessed the FAT and CAT in correcting anterior open bite and in controlling the vertical dimension.*	FAT (*n* = 17), 4 had TAD for molar intrusion
5-Gaffuri et al. (2020) (148)	Prospective Cephalometric study	Invisalign G6	24 adults Class I or Class II, severe crowding or bimaxillary malocclusion, all had 4 first premolars extracted CAT (*n* = 12) (ANB*°* = 2 ± 4) Had 35 (±5) pairs of upper and lower aligners and no IPR, power arms were added to the upper and lower canines for attachment of elastics to the first-molar buttons. Two weeks wear protocol *Assessed effectiveness of Invisalign and fixed appliances in first-premolar extraction cases using ABO Objective Grading System and ABO standard cephalometric analysis.*	FAT (*n* = 12) (ANB*°* = 2.2 ± 2.1)
6-Steele et al. (2022) (20)	Retrospective study	Invisalign	53 adults AOB cases with crowding <6 mm CAT (*n* = 29) *Assessed the dentoskeletal effects of clear aligners (Invisalign) vs. miniplate-supported posterior intrusion and identify factors associated with posttreatment overbite in adults with AOB.*	FAT (*n* = 24) and miniplate-supported posterior intrusion
7-Lin et al. (2022) (58)	Randomised Clinical Trial	Invisalign	54 adults Class I molar and canine relationships, non-extraction, mandibular crowding <4 mm and no missing teeth CAT (*n* = 26) *Assessed the treatment and post-treatment effects of Invisalign aligners (SmartForce) and attachments to traditional fixed appliances.*	FAT (*n* = 28)
8-Frenkel et al. (2024) (56)	Retrospective study	Invisalign	CAT (*n* = 44) Age range = 12–56 Class I malocclusion, non-extraction *Assessed the changes in occlusal contacts.*	FAT (*n* = 36) Age range = 12–23
9-Song et al. (2024) (68)	Retrospective study	Invisalign	57 adults Angle Class I molar relationship with bialveolar protrusion, arch length discrepancy ≤4 mm, had 4 first premolar extractions, required maximum anterior teeth retraction CAT (*n* = 27) *Assessed the positional (overbite and interincisal angle, tipping) changes after CAT and FAT with 4 first premolar extractions.*	FAT (*n* = 30), 0.022″, Roth prescription
10-Gaikwad et al. (2025) (67)	Randomised Clinical Trial	Invisalign	112 adults Mild-to-moderate malocclusion (Angle Class I or Class II division 1), non-extraction or extraction of maximum two premolars per quadrant CAT (*n* = 55) *Assessed the post-treatment occlusal outcome using PAR index*.	FAT (*n* = 58), 0.022″, MBT prescription
11-Al-Somairi et al. (2025) (105)	Retrospective CBCT study	Invisalign	120 adults Class I malocclusion, ≤7 mm crowding, undergoing non-extraction or extraction (four first premolars) CAT (*n* = 60) Non-extraction (*n* = 30) or extraction (*n* = 30) *Assessed the three-dimensional alveolar bone changes (alveolar bone thickness, height, root length, and bone defects) and root resorption in skeletal Class I malocclusion under extraction and non-extraction protocols.*	FAT (*n* = 60) Non-extraction (*n* = 30) or extraction (*n* = 30)
12-Oliva et al. (2026) (112)	Retrospective study	Invisalign	24 adolescents CAT (*n* = 12) Skeletal Class I (50%), Class II (50%) OJ = 3.1 ± 0.8, OB = 3.1 ± 1.2 *Assessed the effectiveness of CAT and FAT in establishing adequate occlusal contacts.*	FAT (*n* = 12) Skeletal Class I (33.3%), Class II (66.7%) OJ = 3.1 ± 1.3, OB = 3.3 ± 1.2
13-Pham et al. (2026) (103)	Randomised Clinical Trial, Cephalometric analysis	Invisalign	CAT group (30?) *Assessed the efficacy of deepbite correction with ANB angles between −4° and 8°.*	FAT (n not specified)
14-Galán-López et al. (2026) (94)	Prospective cephalometric cohort study	Clear aligners	52 adults CAT (*n* = 26), 16 Class I, 8 Class II, 2 Class III *Assessed vertical dimensional changes in mild-to-moderate malocclusions.*	FAT (*n* = 26), 17 Class I, 7 Class II, 2 Class III

Narrative reviews and opinion papers, being among the weaker forms of evidence synthesis, were also considered when discussing the findings, particularly to identify emerging clinical patterns, and highlight areas where primary evidence remains sparse, but were not used as the primary basis for clinical conclusions.

There were five studies reporting using Angel aligner (95), F22 aligners (102, 144), Nuvola® aligners (145), Smartee® system (120), and another four studies with unspecified aligner brands (43, 94, 96, 110), severely limiting generalizability.

Virtual treatment planning software, such as ClinCheck (Align Technology), predicts tooth movements but frequently diverges from achieved clinical outcomes. Reviewing the available studies, comparisons between predicted and actual outcomes mainly rely on intraoral scans, and sometimes CBCT (*n* = 10) (36, 43, 87, 95, 105, 109, 110, 137, 140, 143) which is preferred for its accuracy, or cephalometric analysis (*n* = 7) (19, 22, 24, 28, 94, 103, 148). A preliminary CBCT-based retrospective study (109) provided three-dimensional evaluation of aligner predictability for maxillary transverse and anteroposterior movements, exemplifying the methodological advantage of volumetric imaging over surface scan comparisons alone.

Intra-oral scans employing three-dimensional (3D) metrology (2) use stable anatomical landmarks, such as the palatal vault or bilateral mandibular lingual tori in the premolar regions; however, not all scans capture these landmarks accurately. The muco-gingival junction has also been proposed as a reference for comparison (9). Most studies are retrospective, often conducted by a single clinician, and variations in treatment strategies among clinicians may introduce systematic errors. Additionally, studies predominantly focus on Invisalign (8, 10), with some reporting on initial set of aligners rather than completed cases, and face challenges in quantifying biomechanics and root lengths. These limitations underscore the need for prospective studies with standardized CAT protocols that specifically report on the use of auxiliaries (e.g., elastics), temporary anchorage devices (TADs), wear protocols (e.g., one- or two-week regimens), presence of over-correction, interproximal reduction (IPR) and number aligners including the revisions. IPR plays a great role in CAT in particular for correction of asymmetric dental relationships (10). Literature indicates that aligner therapy is closely associated with IPR, which should ideally be performed accurately and measured with a gauge. However, evidence suggests that orthodontists may underperform planned IPR by up to 56% (12). Studies are also deficient in utilizing subgroup analyses to report on auxiliaries, overcorrections, refinements, and patient compliance. Factors mentioned increase the risk of biases, confounding variables, and difficulties in establishing causality in CAT research (8, 10, 11).

The substantial heterogeneity of the included evidence base warrants explicit acknowledgement before interpreting any pooled or aggregate figures. Included studies varied across multiple dimensions simultaneously: participant age group (adolescents vs. adults), treatment protocol (extraction vs. non-extraction), aligner generation and brand, attachment design and protocol, outcome measurement methodology (intraoral scan superimposition, CBCT volumetric analysis, or 2D cephalometric analysis), wear schedule, and target malocclusion type. Pooling conclusions across such heterogeneous evidence risks oversimplification and may obscure clinically important subgroup differences, for example, between adolescents and adults, or between extraction and non-extraction cases. Accuracy figures presented in the following sections should therefore be understood as approximations within their respective methodological contexts and interpreted at a subgroup level wherever the available data permit.

### 3.2 Prediction vs. achieved outcome

A methodological caveat is necessary when interpreting the accuracy percentages cited throughout this review. Different studies employed different measurement approaches, intraoral scan superimposition, CBCT volumetric analysis, or 2D cephalometric measurement, using different reference landmarks, software platforms, superimposition techniques, and definitions of “accuracy.” Direct numerical comparison of percentages across studies (e.g., equating a 50% figure from an intraoral scan study with a 50% figure from a CBCT study) is therefore potentially misleading. All figures presented below should be understood as approximations within their original methodological contexts, not as interchangeable benchmarks.

Based on early-generation Invisalign studies, a mean accuracy of approximately 50% is reported; newer materials (EX30, SmartTrack) and other aligners may differ, but data are limited (13, 14). A scoping review concluded that most CAT studies (76%) were designed as cohort studies and majority included only non-extraction treatments (73%), and 79% reported results achieved with the Invisalign system (10). This clinical finding implies that orthodontists should anticipate under-expression of planned movements and incorporate over-corrections into treatment plans. Even with limited-stage systems such as Invisalign Lite, a retrospective analysis demonstrated significant discrepancies between planned and achieved overjet, overbite, arch depth, and incisor inclination changes, suggesting that predictability challenges are not confined to full comprehensive aligner protocols (101). In extraction cases, upper first molars exhibit greater mesial tipping, translation, and intrusion than predicted, while central incisors demonstrate less retraction, increased lingual crown torque, and extrusion (15). Rotational movements, particularly for canines, are notably inaccurate, as evidenced by a systematic review of six studies (16).

### 3.3 Bite-block effect of aligners

Vertical control in CAT differs from fixed appliances, which tend to extrude posterior teeth and worsen the anterior open bite (17, 18). Talens-Cogollos et al. (19) report that aligners promote posterior intrusion of approximately 1 mm in Class I malocclusions, potentially increasing overbite or causing bilateral posterior open bite (20). A prospective cephalometric cohort study (94) further confirmed differential vertical dimension changes between CAT and FAT, supporting the notion that the two systems have distinct vertical effects on dentoskeletal structures. Due to the bite-block effect of aligners, CAT is more effective in increasing overbite (e.g., AOB correction) than reducing it (e.g., increased OB correction) (5).

### 3.4 Anterior open bite (AOB) correction

For AOB correction, CAT achieves 58.3%–90% of planned changes, primarily through incisor extrusion and minor posterior intrusion (5, 21–24). Overall, extrusion of maxillary central incisors was more accurate than intrusion (8, 14), with anterior teeth extrusion accuracy ranging from 29.6% to 56.4% (13, 14). Studies report mean corrections of 3.3–3.4 mm in non-extraction adult patients (19–24), with variable molar intrusion patterns, including primarily maxillary (24), mandibular (18), or both (23). Posterior bite-block attachments (i.e., thickened posterior aligner areas, distinct from tooth attachments) have questionable efficacy, as AOB closure primarily results from anterior extrusion (25). In terms of aligner change intervals, two-week wear protocols demonstrate greater effectiveness and predictability (0.49 mm greater AOB closure) compared to one-week protocols (25, 26).

### 3.5 Deep bite (overbite) management

Deep bite management with CAT showed limited success, with overbite reductions ranging from 1.5–3.3 mm and accuracy of 28%–55% (27–35). Anterior intrusion accuracy ranges from 33.4–53.3% (8, 13, 14), with higher accuracy in adolescents (63.5%, 1.7 mm) compared to adults (45.3%, 0.9 mm) (30). Wu et al. (110) retrospectively assessed the intrusion of lower incisors in deep overbite patients in the mixed dentition (average age = 9.2 years) and reported accuracy ranges of 33.3% to 34.6%. However, a recent study (89) reporting in both adult and paediatric populations using Invisalign reported the mean achieved bodily intrusion was only 9.5% of the amount predicted by ClinCheck software which is strikingly lower figure than the 33%–53% range commonly cited in the literature*.* According to a cephalometric study by Khosravi et al. (28), overbite correction (1.5 mm) mechanisms included mandibular incisor proclination, maxillary incisor intrusion, and mandibular molar extrusion. Shahabuddin et al. (119), reported mean overbite correction of 33%, with a 1.15 mm improvement in adult deepbite patients after the first set of aligners, suggesting overcorrection and refinements are needed in most patients with a deepbite. However, only 28%–55% of predicted overbite reduction is achieved in non-extraction adult cases (8, 27–34), and even less (8.69%) in extraction cases (5), suggesting that a hybrid approach combining CAT with fixed appliances may enhance overbite reduction in extraction cases.

Bite ramps, added to the palatal surface of upper aligners in incisor area (analogous to functional anterior bite planes), achieve approximately 43.4% reduction accuracy but do not significantly improve overbite reduction predictability, compared to treatment protocol without Bite ramps (39.2%) (32), potentially due to posterior occlusal coverage limiting eruption (33). Suggestions regarding bite ramp or bite plane efficacy are derived from a small number of studies (32, 33) and should be applied with caution; however, aligns with the fact that coverage of posterior teeth prevent their eruption that help in overbite correction. Stiffer aligner materials (e.g., EX30) outperform more flexible materials (e.g., SmartTrack) in overbite reduction (32, 35). Overall accuracy of posterior teeth extrusion (premolars and molars), which is part of the overbite correction, was 35.1% to 63.1% (8, 14). Finite element analysis (106) explored how aligner thickness (0.75, 0.95 and 1.15 mm) and attachment types (no attachment, horizontal attachment, occlusally bevelled horizontal attachment) interact to determine the mechanical effectiveness of posterior intrusion forces, suggesting that the combination of 1.15 mm aligner thickness with occlusally bevelled horizontal attachment represents the optimal configuration for posterior intrusion. The most recent study by Kou et al. (120), used Biteplanes incorporated in CAT in a retrospective study of adult cases with deepbite, If BPs were utilized, they were placed on canines, incisors, or both based on the anterior overjet to ensure contact with the lower anterior teeth. Second, over-correction was incorporated for anterior deep overbite. Typically, the anterior overbite was designed to an edge-to-edge or a 1–2 mm open bite, based on the initial severity of the deep overbite.

The study by Jin et al. (34) utilized an “over-correction protocol” in patients with hypodivergent or average vertical skeletal patterns (FH-MP < 32°) and a mean initial overbite of 4.6 mm. This protocol incorporated “reverse curve of Spee mechanics” in the aligners, including a deepened maxillary occlusal curve and premolar attachments. The simulation included 2.5–3.0 mm of over-expressed intrusion (i.e., increased premolar extrusion and incisor intrusion, particularly for upper and lower incisors, with minimal effects on canines) (34). This approach achieved 3.3 mm overbite reductions (52.2% of planned) (34). The study suggested that greater proclination of upper incisors (SN-U1) and a more pronounced Class II skeletal base (ANB°) reduce incisor intrusion efficacy, as retroclination of maxillary incisors to correct Class II sagittal discrepancies often results in incisor elongation, compromising intrusion effectiveness (34). A recent study of adult female patients who underwent non-extraction CAT without planned overcorrections concluded that the average accuracy of intrusion for deep overbite correction in the first phase (first set of aligners) was 45.54% (36). Assessing the CBCT in 9 completed cases who had refinement aligners, the average correction for deep overbite was 41.18%.

The most recent systematic review concluded that CAT is effective for correcting mild to moderate dentoalveolar deep bite (ranging from 0.4 mm to 3.8 mm), with accuracy varying between 33% and 48.88% across studies (37). For skeletal cases CAT efficacy compared to full-fixed appliance (FFA) remains uncertain. They also suggested that the planned correction of deep bites with CAT often falls short of the actual achieved correction, making overcorrection or refinements necessary (37).

### 3.6 Overjet correction, curve of spee levelling and posterior extrusion

Overjet correction with CAT achieves approximately 50% of predicted outcomes, with planned increases in overjet (e.g., Class III correction) being more accurate than decreases (e.g., Class II correction) (5). In adolescent patients, Class II correction achieved through CAT combined with intermaxillary elastics has been retrospectively evaluated, with outcomes highlighting both the potential and limitations of elastic auxiliaries in sagittal correction with aligners (97). Approximately one-third of the planned overjet reduction and just less than half of the planned intermaxillary sagittal FPM changes were achieved with an initial series of Invisalign aligners and Class II elastics in a sample of adolescent patients (97). Taffarel et al. (136) assessed whether the CAT of Class II malocclusion with distalization of posterior teeth would meet the ABO Model Grading System, comparing the final ClinCheck and post-treatment results, the ABO Score showed significant difference between the predictions and results for alignment and rotation, buccolingual inclination, OJ, OB, occlusal contact, occlusal relationship, and molar relationship. The final score of the ABO scores did not meet the standards for Class II correction, contrary to what the ClinCheck Pro software predicted.

Treatment outcomes in the adult with Class II subdivision malocclusion treated with CAT were also examined retrospectively by Hatami et al. (107). On average, 36.8% of the planned molar relationship correction and 23.8% of the planned midline correction were achieved. Surprisingly, overjet increased rather than decreased (107), highlighting limited predictability in achieving unilateral Class II molar correction, overjet reduction, and midline improvement in Class II subdivision malocclusions (107).

The retrospective study by Goh et al. (38) initially reported the mandibular Curve of Spee (CoS) leveling accuracy is approximately 35% of predicted, whereas a recent systematic review reported a CoS correction with a varied predictability from 35% to 72% (93). A recent retrospective study (92) compared 2 groups of 30 patients who received CAT with Invisalign, authors reported that the extraction group showed less effectiveness in leveling the CoS compared to the non-extraction group (92). Planned posterior intrusion in the maxilla is more accurate for first molars (117% expression) but less accurate for extrusion of premolars, with mid-arch movements demonstrating under-expression ranging from −14% to −48% (39). This highlights the need for overcorrection using reverse CoS mechanics in aligners (34).

### 3.7 Rotational movements

Overall rotational accuracy based on 12 studies included (1 RCT, 11 non-randomised); wide heterogeneity in aligner systems and tooth types, ranged from 36% to 85%, averaging approximately 65%, while canines and premolars remain challenging (8, 16, 85). In particular, for canine and premolar the accuracy was 35.8%–55.2% (8, 14, 40) and 39.2%–67.0% (8, 14, 16), respectively. Rotational movements exhibit a shortfall of approximately 5° between planned and achieved outcomes (41, 42). Regarding attachment design for rotational correction, a retrospective study by Issa et al. (96) found differences in effectiveness between single and double attachment configurations for premolar rotation correction in adults, suggesting two attachments may influence rotational outcomes positively.

Root torque expression with CAT is challenging. Planning anterior lingual crown inclination or labial root inclination is more accurate (4). A CBCT study by Jiang et al. (43) found that pure tipping (72.5%) is the most predictable movement, while torque (35.2%) is the least predictable. Labial root movement is significantly more predictable than lingual root movement, and labial movement of mandibular incisors is more achievable than that of maxillary incisors (35). Torque is generally under-expressed for labial movements and fully or over-expressed for lingual movements, indicating CAT limited efficacy in labial incisor torque (35, 44). Incisor torque expression shows mean accuracies of 41.9% for maxillary incisors and 58.2% for mandibular incisors (45, 46). Attachments on lower incisors improve torque expression predictability (47).

### 3.8 Root paralleling and arch expansion

Achieving root paralleling in extraction cases is challenging and necessitates attachments. Root angulation at extraction sites shows over-expressed divergence and under-expressed convergence (48, 49). Feng et al. (140) concluded that the anti-tipping design, distal crown tipping of posterior teeth and mesial crown tipping of canines, could avoid unwanted tipping toward the extraction space during space closure in adults who had premolar extraction.

Houle et al. (50) reported an arch expansion accuracy of 72.8% for the maxilla and 87.7% for the mandible, with overestimated bodily movement often resulting in tipping (i.e., more tipping and less root movement than planned) (50). Another study (51) of patients with transverse malocclusions (i.e., posterior crossbites) revealed that predicted (virtual planning) expansion were greater than achieved: a mean of 0.63 mm more expansion at the canine level, 0.77 mm at first premolar, 0.81 at second premolar, 0.69 mm at first molar, and 0.25 mm at second molar. All predicted expansions, apart from the second molar, were significantly higher than the actual outcomes (51). Further, analyzing a cohort of patients with a mean age of 17 ± 3.2 years old demonstrated that the overall accuracy of the planned expansion (between 1.6 mm and 3.5 mm) was 70%, regardless of tooth type (52). The accuracy of the gingival level expansion was lower, around 50%, compared to the cusp level expansion, where the accuracy was between 70% and 82%. In the inter-cusp measurements, the expansion was more accurate for the first premolar (93.53%) and less for the first molar (70.55%). In a mixed dentition study (53), the predictability of expansion did not exceed the 50% that reached 70% after refinement. All mentioned studies used the Invisalign system (50–53). A recent systematic review and meta-analysis (98) corroborated these findings, confirming that predicted transversal expansion consistently exceeds achieved outcomes with Invisalign aligners in permanent dentition. Zhou et al. (137) assessed the efficiency of bodily expansion in upper arch expansion using CBCT in 20 adults and concluded that CAT could increase the arch width, but expansion was achieved by tipping movement. A recent CBCT study of orthodontic tooth movement using CAT showed that transverse movements at the canines are more predictable than at premolars and molars (109). There were significant mean differences in the transverse dimension between the actual and predicted movements at the crown level for the first molars and first premolars and root level, 1.88 mm, 0.64 mm, 5.50 mm, and 3.10 mm, respectively (109), indicating more crown tipping than root expansions.

### 3.9 Posterior occlusal contacts

CAT results in fewer posterior occlusal contacts than planned, fewer than fixed appliance therapy, and fewer than pre-treatment conditions (31, 54–56). Posterior open contacts are common post-CAT, partly due to poor torque control and increased aligner thickness contributing to posterior open contacts (56). A review (99) further elaborated on the aetiology, clinical management, and prevention of posterior open bites generated during CAT, providing practical guidance for clinicians encountering this complication. A recent retrospective study (111) further characterised differences in the distribution and quality of occlusal contacts obtained with CAT compared to conventional orthodontic appliances, adding to the growing body of evidence that occlusal settling is a consistent challenge following aligner treatment. Over 50% of CAT cases require refinements or fixed adjuncts treatment (57). Vertical settling elastics applied during fine-tuning staging have been proposed as an effective adjunct for resolving posterior open bites that persist at the end of active aligner treatment (95).

### 3.10. Comparison with fixed appliance therapy (FAT)

Several studies and systematic reviews that compared CAT with fixed appliance therapy (FAT) (54, 58–68, 117, 129) were considered for this scoping review. For instance, in mild Class I cases, outcomes were comparable, though CAT requires an additional 4.8 months to complete treatment (58); or in hyperdivergent AOB cases (mandibular plane–SN angle ≥ 38°), AOB correction showed minimal differences between CAT and FAT, but CAT resulted in significantly greater lower incisor extrusion (59). A recent study compared two groups of patients with mild to moderate Class I and Class II division 1 malocclusion who received either CAT or FAT. Approximately one-third of the patients in both groups underwent extractions as part of their treatment. The treatment duration was significantly longer with CAT (18.2 ± 3.1 months) compared to FAT (14.5 ± 2.8 months). However, both groups achieved similar reductions in the Peer Assessment Rating (PAR) index (*p* = 0.082) (67). When CAT (Invisalign) and FAT were compared in patients with bimaxillary protrusion requiring extraction of four first premolars, CAT treatment took six months longer and showed approximately 1 mm greater overbite, more lingual tipping of the maxillary central incisors, distal tipping of the maxillary canines, and mesial tipping of the maxillary first and second molars after maximum anterior retraction compared with FAT *(*68). Jaber et al. (117), in a RCT, looked at 40 adult patients with Class I severe crowding who required four first premolars extraction and randomly allocated into them into two treatment groups: CAT and FAT. Post-treatment records were evaluated using the American Board of Orthodontics Objective Grading System (ABO-OGS) (118). When comparison of the successful cases between the two groups was made, 11 cases received passing scores, and 9 cases received failing scores in the CAT group. Whereas in the FAT group, 17 cases received passing scores, and 3 received a failing score. No statistically significant differences were found in the passing rates between of the CAT and FAT groups (*P* = 0.421), due to probably a power problem, with only 20 patients per group, the study was underpowered to detect a 30% difference in pass rates. The CAT and FAT mean (SD) ABO-OGS scores were 17.50 (7.4) and 12.89 (6.3), respectively. CAT had about 35% lower probability of a passing outcome compared to FAT.

A few systematic reviews investigated comparison of effectiveness between FAT and CAT (60–66, 108, 114). A systematic review confirms CAT's efficacy for mild cases, with FAT demonstrating superiority for severe cases (65). Another systematic review (108) specifically compared CAT and FAT effectiveness for anterior tooth movements in adults, corroborating FAT's advantage for complex anterior tooth control while acknowledging CAT's adequacy for milder presentations. However, recent systematic review of fifteen trials (10 of them were non-extraction cases) involving 1,084 patients concluded that no significant differences were found in treatment quality or duration between CAT and FAT in non-extraction cases, although sensitivity analyses suggested shorter treatment duration with clear aligners (66). However, in extraction cases, based on findings of four studies that used American Board of Orthodontics Objective Grading System, FAT provided superior treatment quality, attributed to enhanced control of tooth movements (66). A randomised clinical trial by Pham et al. (103) added Level I evidence to this area, reporting on both clinical effectiveness and patient satisfaction outcomes for CAT in deep bite malocclusion, with findings broadly consistent with the moderate efficacy described in observational studies. The clear aligner group demonstrated a significantly shorter treatment duration (20.77 ± 6.43 months) compared to the braces group (27.47 ± 4.81 months). There were no significant differences in overbite improvement or overjet reduction between the 2 groups (103). Cephalometric analysis showed that fixed braces had a greater impact on vertical facial dimensions, while clear aligners were associated with increased upper incisor proclination (103). Baneshi et al. (114), concluded that the overall quality of evidence from the included studies in their systematic review was low and both CAT and FAT worked well in treating simple malocclusions treated on a non-extraction basis.

Extraction-based CAT remains poorly characterized in the literature, the recent meta-analysis dedicated exclusively to extraction-based CAT (86) looking into Primary outcomes such as predicted vs. achieved tooth movement and root angulation/parallelism as well as Secondary outcomes: treatment duration and clinical indices (ABO-OGS, PAR). They confirmed what individual studies suggested: fixed appliances provide superior biomechanical control in extraction cases, making hybrid approaches the most defensible clinical consideration (86).

Evidence of post treatment changes and relapse between FAT and CAT is limited. Kuncio et al. (129), using a sample 22 patients (11 CAT and 11 FAT), concluded that patients treated with Invisalign relapsed more than those treated with conventional fixed appliances. Le et al. (58), compared CAT and FAT after 6 months of retention. During the posttreatment period, alignment and overjet worsened significantly in the CAT group, while buccolingual inclinations and occlusal relations improved. For FAT, alignment worsened and buccolingual inclinations improved. There was no statistically significant between CAT and FAT in post-treatment changes of the total OGS scores.

## 4 Discussion

The following clinical observations are derived from evidence mapping rather than formal evidence synthesis. As a scoping review, this work aims to comprehensively map what is known about CAT predictability and identify gaps in the literature, not to generate formal clinical guidelines. Most included evidence is Level III–IV (retrospective cohort studies and expert consensus). Specific quantitative figures cited (e.g., percentage overcorrections, rotation shortfalls) reflect findings reported across individual heterogeneous studies and should not be interpreted as universally validated clinical protocols. In the absence of adequately powered RCTs, all practical guidance presented below represents cautious clinical inference rather than evidence-based directives and should be applied with individual clinical judgment. The inclusion of narrative reviews and opinion papers in this scoping review is consistent with the broad mapping objectives of the methodology; these sources were considered when discussing the findings to provide explanatory context and to surface clinically important observations in movement types with sparse primary evidence. However, to maintain appropriate epistemic rigour, conclusions derived primarily from narrative reviews are described in observational rather than prescriptive terms, and are clearly distinguished from findings supported by primary cohort data or systematic review evidence.

### 4.1 Predictability and overcorrections in clear aligner therapy

The less than ideal predictability of CAT (1, 8, 35) underscores the need for planned overcorrections, as certain movements, such as tipping or incisor extrusion, are relatively predictable, whereas overbite reduction, root control, and rotational corrections remain challenging to achieve accurately (1, 8, 35).

Fixed appliance therapy (FAT) achieves a mean overbite reduction of approximately 3 mm, with initial severity influencing long-term stability (69). FAT remains the orthodontic appliance of choice when correcting a deep overbite (70). In contrast, CAT demonstrates limited efficacy in overbite reduction, with accuracies ranging from 28% to 55% (35). Blundell (71), reviewing 37 studies, concluded that <50% of the planned overbite reduction is expressed when using CAT. Molar extrusion remains one of the least accurate movements with CAT, with 30%–40% expression of the planned movement. Contrary to FAT, deep bite correction with CAT is primarily achieved through maxillary and mandibular incisor intrusion (72). Incisor intrusion for overbite correction requires anchorage from adjacent teeth and typically necessitating 50%–100% over-treatment (1, 2). This involves incorporating 2.5–3.0 mm of “over-expressed intrusion” and employing “reverse CoS mechanics” (e.g., increased premolar extrusion) in the digital treatment setup to address excessive overbite (1, 34). For instance, while the ideal overbite is approximately 2 mm, the finishing setup should target 0 to −2 mm, or greater in cases with significant occlusal tooth wear (1). Other recommended strategies include using stiffer aligner materials (e.g., EX30), auxiliary vertical elastics to facilitate posterior extrusion, conventional posterior horizontal attachments, slower aligner change intervals (e.g., two-week protocols), and precise interproximal reduction (IPR) monitored with a gauge (1, 2, 8, 35). A randomised clinical trial (104) further evaluated the effect of different Invisalign wear time protocols on overall treatment effectiveness, providing RCT-level evidence that wear duration is a meaningful clinical variable influencing orthodontic outcomes. The 12 h daily wear protocol achieved adequate alignment in the maxilla in cases of very mild crowding. However, 22 h daily wear protocol showed more efficiency in correction of mandibular irregularity (104). Castroflorio et al. (142), concluded that a 7-day protocol is generally sufficient for most movements, but molar torque control, lower canine and bicuspid rotation and torque control and lower molars rotation need 14 days aligner change. Current evidence does not support the routine use of power/torque ridges or anterior bite ramps for overbite reduction (2).

For anterior open bite correction, incisor extrusion is enhanced by conventional or beveled horizontal attachments placed on anterior teeth to facilitate extrusion and on posterior teeth to improve aligner retention and support posterior intrusion (1, 2, 8, 35). Additionally, angled (approximately 45°) “Sash” attachments on posterior teeth can further promote these movements (73). IPR and auxiliary vertical elastics may also be necessary to achieve AOB correction (1).

It is equally important to recognise that deep bite and anterior open bite correction are among the movement categories most prone to under-expression, and clinicians should consistently anticipate the need for overcorrection and sequential refinements in these cases (1, 2). Responsibility for accurate treatment planning and achieving the desired outcome rests with the treating clinician, not with the aligner company or its planning software. Artificial intelligence-assisted planning tools such as ClinCheck (Align Technology) generate simulations that reflect biomechanically plausible, but not clinically guaranteed tooth movements. Software predictions are inherently limited by material properties, individual patient biology, and compliance, and do not account for patient-specific tissue adaptation constraints. Notably, Invisalign's ClinCheck platform now displays predictability ratings indicating the likelihood of achieving individual tooth movements, offering clinicians an explicit visual indicator of movement difficulty. Incorporating these predictability estimates into the treatment planning process, and informed consent discussions may help set realistic expectations for both clinicians and patients.

### 4.2 Anterior open bite management

Anterior open bite (AOB) can adversely affect oral function, aesthetics, and psychosocial well-being, with prevalences ranging from 0.4% to 16.5% in orthodontically untreated individuals (74, 75). CAT demonstrates greater accuracy in achieving incisor extrusion and molar intrusion compared to incisor intrusion and molar extrusion (8, 14, 35, 75), indicating that aligners are more effective for anterior bite closure than bite opening (8, 35, 71, 76). In the context of retention provisions after treatment of AOB with CAT, Suh et al. (77) reported that combined maxillary and mandibular fixed retainers with vacuum-formed retainers provided good short-term stability (2.1 ± 1.1 years) for AOB treatment, with no significant changes in cephalometric measurements. However, in AOB cases, clinicians must assess whether increased incisor and gingival exposure is aesthetically acceptable (78). Gingival exposure exceeding 3 mm is generally considered unaesthetic, often requiring surgical correction (79) or combined use of vertical anchorage (e.g., temporary anchorage devices [TADs]) with FAT or CAT (78). In patients with dental protrusion, IPR is frequently employed as a strategic adjunct to facilitate incisor retraction, torque correction, and relative incisor extrusion (78). CAT is most effective for managing AOB of less than 3 mm, particularly when incisor extrusion is an acceptable treatment objective (78).

### 4.3 Biomechanical considerations

From a biomechanical perspective, rotational movements require a couple or moment to rotate the tooth around its long axis, necessitating attachments (1). Both rotational and torque movements typically require 5°–15° of over-correction, staged at approximately 1° per aligner (1, 2). Data on rotational control are limited; larger attachments with defined edges demonstrate superior performance, while dual-surface (buccal/lingual) or adjacent tooth attachments provide no clear benefit (80). Arch expansion typically requires 1–2 mm of overtreatment and the use of attachments to enhance root control (1, 2). To promote improved occlusal contact and natural settling, the immediate use of thermoplastic retainers following CAT is not recommended (2). There is tendency to prefer non-extraction treatment that is based on arch expansion, a retrospective study showed that non-extraction CAT was associated with increased presence of alveolar bone dehiscence and fenestration (87); Thus, root-bone relationship should be considered and evaluated carefully, especially when arch expansion is planned (88). Furthermore, a comparative CBCT study (105) in Class I skeletal malocclusion treated with FAT or CAT, under extraction and non-extraction protocols, showed that FAT resulted in more pronounced alveolar bone remodeling and root resorption compared to CAT, most notably in extraction cases. While CAT helped in preserving labial bone and minimizing root resorption, neither method completely averted bone loss (105).

From a safety perspective, recent systematic review evidence indicates that root resorption occurs with both CAT and fixed appliances, and the meta-analysis found that CAT caused significantly less external apical root resorption than FAT for all tooth types except for mandibular lateral (111).

### 4.4 Attachments and treatment strategies

Rigid plastic templates that provide optimal precision for attachment placement, combined with high-filler content composites for bonding and attachments with angled edges to facilitate tooth movement, are recommended (81, 82). A finite element analysis (100) highlighted that flat-shaped attachments (rectangular and trapezoidal) generated the greatest crown displacement but induced higher PDL strain and localized bone stress, particularly at the root apex and alveolar crest. Curved attachments; however, provided more diffused load distribution but significantly reduced movement efficiency. Posterior attachments enhance anchorage, though evidence supporting their efficacy for intrusion or extrusion is limited (80). A very recent systematic review on clear aligner attachments concluded that current evidence is mostly *in vitro*-based, demanding more clinical studies to explore their effectiveness (127).

Limited evidence from a review of 500 Invisalign cases suggests that treatment duration is approximately 2 years (similar to FAT), with patients requiring an average of 2.5 refinement phases and 17.2% needing supplementary FAT. Meade et al. (4), reviewing 324 Invisalign cases from 11 orthodontist reported that most (99.4%) required a refinement phase with a median of 2 (interquartile range = 2, 2–7) refinement plans recorded. These findings underscore the importance of considering the need for refinements, incorporating realistic treatment timelines, and the possibility of conversion to FAT into the informed consent process (83). Kravitz et al. (83) evaluated the conversion rate to fixed appliances and reported that approximately 17.2% of adult patients treated with CAT were switched from Invisalign to FAT, highlighting the lower predictability of CAT in the treatment of certain malocclusions (1, 3–5, 8, 14). The average number of aligners used in this group was 80.6, followed by an additional 6.9 months of fixed appliance treatment, compared with 64.1 aligners in patients who completed treatment using CAT alone. These findings further suggest that patients who choose CAT should have realistic expectations and be prepared for two to three refinement phases, with the possibility that supplementary FAT may also be required. Evidence suggests that CAT is less predictable for certain types of tooth movement, including deep bite correction (incisor intrusion), rotations, closure of extraction spaces, alignment of impacted teeth, expansion, and sagittal correction (1, 3–5, 8, 14). Hybrid approaches that combine CAT with fixed appliance therapy (FAT), whether at the beginning or at the end of CAT, may be appropriate for managing impacted teeth, rotations, closure of extraction spaces, or severe malocclusions (5, 7, 84). Supporting this clinical consideration, Cremonini et al. (102) demonstrated in a retrospective cohort study that a hybrid CAT-fixed approach (CAT combined with a segmental lingual appliance to correct rotation without attachments) improved accuracy in managing severe rotations of rounded teeth that are particularly resistant to pure aligner correction. Limitations of existing studies include retrospective biases, a predominant focus on Invisalign, and patient compliance issues, with only 37% of patients wearing aligners for more than 22 h per day (12). Future research should prioritize prospective study designs, evaluate diverse aligner systems, investigate the role of TADs, and assess long-term stability to elucidate the impact of attachment number, size, shape, and position on specific orthodontic movements (80).

The suggestion to avoid immediate thermoplastic retainers post-CAT to facilitate posterior settling is based on limited evidence (2). Given the known risk of relapse, particularly in cases of AOB, rotational correction, and expansion, clinicians should weigh the potential benefit of posterior settling against the risk of treatment relapse. Individualized retention protocols are recommended, and this guidance should not be applied uniformly without consideration of case-specific relapse risk. More broadly, clinical considerations concerning bite ramp designs, trimline configurations, attachment geometries, and retention strategies presented in this review are often based on limited primary evidence, in some cases a single retrospective or laboratory investigation, and should be interpreted as preliminary clinical observations rather than validated protocols.

It is worth mentioning a three-round modified Delphi study (90) involving 36 international experts aimed to reach consensus (≥70% agreement threshold) on clinical and extra-clinical aspects of CAT. Consensus reached that CAT achieves outcomes comparable to fixed appliances in Class I, non-extraction, mild-to-moderate crowded cases (90). Expert panel agreed that more RCTs are urgently needed to validate CAT protocols and refine treatment guidelines (90).

The rapid growth of CAT-related publications is itself a meaningful indicator of the field's momentum. A recent bibliometric and network analysis of pivotal aligner literature documented a marked and sustained increase in the number of CAT-related publications over the past decade, reflecting the high clinical and scientific interest in this area (121). This publication growth underscores the urgent need for methodologically robust prospective studies to complement the predominantly retrospective evidence base.

The authors acknowledge that, given the predominance of Invisalign-focused research in North American and Australian literature, contributions from other aligner companies may be underrepresented in this review and needs further investigations to address this geographic and institutional bias in the evidence base.

### 4.5 Evidence gaps and future research direction

Of the 86 primary studies included, 70 (81%) were retrospective, 12 (14%) were prospective, and only 4 (5%) were RCTs. Thus, despite addressing clinically important questions, the available evidence is largely based on lower levels of the conventional evidence hierarchy. Nevertheless, these primary studies have generated 33 secondary publications, including systematic reviews, meta-analyses, narrative reviews, and consensus documents. This imbalance reflects the concern raised by Papageorgiou and Eliades that orthodontics may be producing systematic reviews more rapidly than the high-quality clinical trials needed to support them (150). As Bazargani and Cobourne (151) have argued, combining low-quality primary studies may create an illusion of precision and certainty that is not justified by the underlying evidence. In the case of CAT, evaluating over-correction strategies under routine clinical conditions is inherently difficult to investigate through randomised trials because of ethical and practical barriers to withholding or randomising treatment protocols in patients seeking care. Consequently, much of the current evidence is derived from retrospective studies, predominantly involving a single aligner system (Invisalign), with subsequent conclusions based largely on evidence synthesis. These findings should therefore be interpreted with appropriate caution, recognising the limitations of the underlying evidence rather than the apparent strength suggested by the growing number of publications.

In keeping with scoping review methodology, no formal quality appraisal of included studies was performed. This scoping review is useful for answering much broader questions of “what is the nature of the evidence for effectiveness and accuracy of CAT?” (116). Methodological limitations of included studies are discussed narratively, but no summary measures, assessment of risk of bias and additional analysis were provided (116). The omission of a formal risk-of-bias tool is consistent with scoping review methodology as described by Levac et al. (115) and the PRISMA-ScR guidance (116), which do not mandate quality appraisal. Nonetheless, readers should interpret all quantitative estimates presented in this review considering the pervasive methodological limitations of the included studies, particularly the predominance of single-clinician retrospective designs, small sample sizes, Invisalign centric, mild malocclusion and non-extraction treatment dominance, absence of compliance data, and limited control for treatment auxiliaries. Narrative reviews and opinion papers were also considered when discussing the findings, particularly in sections where primary clinical evidence is sparse, and their lower evidence hierarchy should be borne in mind when appraising the confidence of specific conclusions. To contextualise this: narrative reviews and opinion papers were considered when discussing the findings, primarily to provide mechanistic explanation and clinical framing in areas where controlled primary evidence is limited. However, findings derived from narrative reviews were not used to formulate quantitative conclusions, and readers should weight these contributions accordingly in their appraisal of the evidence.

The present scoping review specifically aimed to identify evidence gaps. The findings of the present scoping review highlighted the need for studies of non-Invisalign systems (Angel, ClearCorrect, Spark, in-office systems, etc.), RCT comparing different attachment morphologies/materials, long-term stability data (>2 years post-CAT), sub-group analyses by compliance, prospective RCTs, and hybrid (TAD/FAT) use. A further limitation is that screening and eligibility assessment were conducted by a single author, which increases the risk of selection bias compared with dual-reviewer designs. This was partially mitigated by conducting two independent screening rounds separated by a two-week interval and by applying a standardised eligibility form throughout.

A structured literature search was conducted in PubMed and Scopus. These databases were selected for their comprehensive coverage of dental and orthodontic literature. The authors acknowledge that additional databases, including Embase, Web of Science, and the Cochrane Library were not searched. This represents a methodological limitation of the present review and may have introduced publication bias by missing relevant studies indexed outside PubMed and Scopus, however high initial number of identified articles suggest that we covered the core of the literature. Future updates to this review should adopt a multi-database search strategy to minimise this risk.

### 4.6 In-office and non-proprietary aligner systems

A notable limitation of the present review is the near-exclusive focus on proprietary aligner systems, particularly Invisalign. In-office or in-house Clear Aligner (CA) fabrication using direct-print or thermoforming workflows, employing biocompatible resins or comparable materials, represents an emerging area of clinical practice that bypasses the external laboratory and company infrastructure required by systems such as Invisalign (122–125). These in-office systems, e.g., Dental Clear LT, Graphy TC-85, offer potential advantages including reduced cost, faster turnaround, and greater clinician control over staging and biomechanics. This is particularly important when a small number of aligners in short term are used as pre-operative stage in restorative and aesthetic dentistry (126). However, peer-reviewed evidence on the accuracy, predictability, and clinical outcomes of in-office aligner systems remains extremely limited, constituting a significant gap in the current literature. Sachdev et al. (141), reported overall CAT tooth movement accuracy of 56.18% for in-house anterior clear aligners with crowding not exceeding 4 mm per arch, non-extraction cases, with no changes in posterior relationship. Jaber et al. (146), reported on a 2-arm parallel RCT comparing in-house CAT and FAT on adults with Class I skeletal and dental malocclusion and severe crowding (>6 mm) who required orthodontic treatment with four first premolars extraction. The findings for both groups were not statistically different for changes in PAR, Little's irregularity index (146). Azevedo et al. (149), reported on dental arch expansion with in-house CA, using 6 aligners in 6 weeks, reporting short-term in-house CA expansion was more effective in premolars than in canines or molars and maintained vertical control. The scarcity of evidence for in-office systems should itself be considered a limitation of this review and a priority area for future investigation.

## 5 Conclusions

This scoping review maps the current evidence on CAT predictability, identifying key gaps including the underrepresentation of non-Invisalign systems, the scarcity of prospective RCTs, and the absence of long-term stability data. These gaps should inform the design of future research.

CAT offers effective treatment for mild-to-moderate malocclusions, with historical and technological foundations enabling precise digital planning. However, achievements often fall short of predictions (approximately 50% accuracy in early-generation studies), and the overcorrection strategies described in this review, while evidence-informed, are derived predominantly from retrospective, Invisalign-focused research and should not be applied as validated clinical benchmarks without further prospective confirmation. Clinicians should anticipate under-expression in rotations, torque, and vertical movements, prioritizing evidence-based strategies for optimal outcomes. While comparable to fixed appliances in simple cases, CAT's limitations in complex scenarios highlight the value of hybrid therapies. Future research should specifically include non-Invisalign systems, particularly newer proprietary materials and attachment designs, to improve generalizability of accuracy data across the full CAT landscape.
